# Next-Gen Neuroprotection in Glaucoma: Synergistic Molecules for Targeted Therapy

**DOI:** 10.3390/jcm14176145

**Published:** 2025-08-30

**Authors:** Alessio Martucci, Massimo Cesareo, Maria Dolores Pinazo-Durán, Francesco Aiello, Giulio Pocobelli, Raffaele Mancino, Carlo Nucci

**Affiliations:** 1 Ophthalmology Unit, Department of Experimental Medicine, University of Rome ‘Tor Vergata’, Montpellier 1, 00133 Rome, Italy; massimo.cesareo@uniroma2.it (M.C.); francescoaiello@hotmail.com (F.A.); mancino@med.uniroma2.it (R.M.); nucci@med.uniroma2.it (C.N.); 2 Ophthalmic Research Unit “Santiago Grisolia”, Foundation for the Promotion of Health and Biomedical Research of the Valencian Community, 46020 Valencia, Spain; pinazoduran@yahoo.es; 3 Department of Ophthalmology, Mid and South Essex NHS Foundation Trust, Southend University Hospital NHS Foundation Trust, Nethermayne, Basildon, Essex SS16 5NL, UK; giulio.pocobelli1@nhs.net

**Keywords:** coenzyme Q10, citicoline, pyruvate, nicotinamide, pyrroloquinoline quinone, homotaurine, berberine, gamma-aminobutyric acid, neuroprotection, glaucoma

## Abstract

Background: Glaucoma is a progressive optic neuropathy marked by retinal ganglion cells (RGCs), apoptosis, vascular insufficiency, oxidative stress, mitochondrial dysfunction, excitotoxicity, and neuroinflammation. While intraocular pressure (IOP) reduction remains the primary intervention, many patients continue to lose vision despite adequate pressure control. Emerging neuroprotective agents—citicoline, coenzyme Q10 (CoQ10), pyruvate, nicotinamide, pyrroloquinoline quinone (PQQ), homotaurine, berberine, and gamma-aminobutyric acid (GABA)—target complementary pathogenic pathways in experimental and clinical settings. Methods: This literature review synthesizes current evidence on glaucoma neuroprotection, specifically drawing on the most relevant and recent studies identified via PubMed. Results: Citicoline enhances phospholipid synthesis, stabilizes mitochondrial membranes, modulates neurotransmitters, and improves electrophysiological and visual field outcomes. CoQ10 preserves mitochondrial bioenergetics, scavenges reactive oxygen species, and mitigates glutamate-induced excitotoxicity. Pyruvate supports energy metabolism, scavenges reactive oxygen species, and restores metabolic transporter expression. Nicotinamide and its precursor nicotinamide riboside boost NAD^+^ levels, protect against early mitochondrial dysfunction, and enhance photopic negative response amplitudes. PQQ reduces systemic inflammation and enhances mitochondrial metabolites, while homotaurine modulates GABAergic signaling and inhibits β-amyloid aggregation. Berberine attenuates excitotoxicity, inflammation, and apoptosis via the P2X7 and GABA-PKC-α pathways. Preclinical models demonstrate synergy when agents are combined to address multiple targets. Clinical trials of fixed-dose combinations—such as citicoline + CoQ10 ± vitamin B3, citicoline + homotaurine ± vitamin E or PQQ, and nicotinamide + pyruvate—show additive improvements in RGCs’ electrophysiology, visual function, contrast sensitivity, and quality of life without altering IOP. Conclusions: A multi-targeted approach is suitable for glaucoma’s complex neurobiology and may slow progression more effectively than monotherapies. Ongoing randomized controlled trials are essential to establish optimal compound ratios, dosages, long-term safety, and structural outcomes. However, current evidence remains limited by small sample sizes, heterogeneous study designs, and a lack of long-term real-world data. Integrating combination neuroprotection into standard care holds promise for preserving vision and reducing the global burden of irreversible glaucoma-related blindness.

## 1. Introduction

Glaucoma is a group of progressive chronic optic neuropathies that lead to characteristic structural damage to the optic nerve head (ONH) and associated loss of the visual field (VF). It is widely recognized as one of the leading causes of irreversible blindness worldwide. The hallmark of glaucoma is the degeneration of retinal ganglion cells (RGCs) and their axons, which ultimately results in loss of vision. Although elevated intraocular pressure (IOP) is an important risk factor and often a central characteristic, glaucoma can also occur with normal IOP, a condition known as normal-tension glaucoma (NTG). Therefore, glaucoma is considered a multifactorial disease, with mechanical, vascular, genetic, and neurodegenerative factors contributing to its pathogenesis [1].

Epidemiologically, glaucoma affects more than 76 million people worldwide. This number is expected to rise to more than 110 million by 2040 due to aging populations and increasing life expectancy. The two most common forms of glaucoma are primary open-angle glaucoma (POAG) and primary angle closure glaucoma (PACG). POAG is the most prevalent type globally, especially in western countries, and is characterized by an open anterior chamber angle with gradual optic nerve (ON) damage and loss of VF. PACG, more common in Asian populations, involves closure of the anterior chamber angle, leading to sudden or chronic increases in IOP and a more rapid progression if not treated promptly. Secondary glaucoma, which occurs due to underlying causes such as trauma, inflammation, or medications, also contributes significantly to the global burden of the disease [2,3].

From a pathophysiological perspective, the most widely accepted mechanisms underlying glaucomatous damage involve mechanical and ischemic stress on the ON. Increased IOP can cause direct mechanical compression of the lamina cribrosa, leading to axonal deformation and impaired axoplasmic flow. This results in the deprivation of neurotrophic factors essential for the survival of RGCs. Furthermore, high IOP can compromise the microcirculation in the ONH, leading to ischemia and hypoxia, further exacerbating neuronal injury. Oxidative stress, mitochondrial dysfunction, and excitotoxicity due to excess glutamate release are also involved in apoptosis of RGCs. Importantly, neuroinflammation and immune system dysregulation are increasingly recognized as contributors to the chronic neurodegenerative process of glaucoma, similar to mechanisms observed in central nervous system disorders such as Alzheimer’s and Parkinson’s diseases [4,5].

Early detection and intervention are critical in the management of glaucoma, as vision loss from this disease is permanent and generally progresses without noticeable symptoms until advanced stages. The primary goal of treatment is to reduce IOP, the only modifiable risk factor currently known to slow the progression of the disease. The reduction in IOP can be achieved through a combination of pharmacologic therapy, laser procedures, and surgical interventions. Topical medications are generally the first line of treatment [6,7]. Combination therapies are often used when monotherapy does not control IOP adequately. In recent years, fixed-dose combination drops have improved patient compliance by reducing the burden of multiple medications [8].

Although IOP reduction remains the cornerstone of glaucoma management, increasing attention is being paid to the concept of neuroprotection. Neuroprotective strategies aim to interrupt the cascade of events that lead to apoptosis of RGCs by targeting oxidative stress, mitochondrial dysfunction, glutamate excitotoxicity, and neuroinflammation [9,10]. However, it is important to note that many putative neuroprotective strategies, including agents such as memantine, have failed to demonstrate efficacy in large clinical trials, warranting cautious interpretation of preliminary findings.

Glaucoma is a complex and multifactorial neurodegenerative disease that poses a major public health challenge due to its asymptomatic onset and irreversible vision loss. Understanding its pathogenesis helps guide a multifaceted treatment approach that includes both IOP-lowering strategies and emerging neuroprotective therapies. Although medications remain central to current management, it can be hypothesized that the use of combined neuroprotective treatments, similar to the approach used with combined hypotensive therapies, may offer greater efficacy in preventing or slowing glaucomatous damage. The objective of this review is to examine the most recent available neuroprotective agents and to summarize current evidence on their combined use.

## 2. Neuroprotective Agents

Several agents with neuroprotective properties have been investigated and, in some cases, introduced into clinical practice. These include compounds with antioxidant, anti-inflammatory, anti-apoptotic, and mitochondrial stabilizing effects. Among the most studied and available on the market are citicoline, coenzyme Q10 (CoQ10), pyruvate, nicotinamide, pyrroloquinoline quinone (PQQ), homotaurine, berberine, gamma-aminobutyric acid (GABA), and vitamins. Although not all are approved specifically as neuroprotective treatments for glaucoma, their mechanisms of action and a growing body of clinical evidence support their potential role as complementary therapies in glaucomatous neurodegeneration [9,11].

### 2.1. Citicoline

Citicoline (cytidine 5′-diphosphocholine) is an endogenous mononucleotide composed of ribose, cytosine, pyrophosphate, and choline, and it serves as an intermediate in phospholipid biosynthesis. Its neuroprotective properties are mediated through multiple mechanisms: increased phosphatidylcholine and cardiolipin synthesis, attenuation of free fatty acid release, and stabilization of mitochondrial membranes. Furthermore, citicoline increases the levels of key neurotransmitters, including dopamine, noradrenaline, serotonin, and acetylcholine, by promoting tyrosine hydroxylase activity and inhibiting dopamine reuptake [11].

Extensive preclinical evidence supports the efficacy of citicoline in cerebrovascular and neurodegenerative conditions. In experimental stroke models, it reduces infarct size, brain edema, and neurological deficits. In Alzheimer’s disease citicoline appears to mitigate β-amyloid accumulation and improve cognitive performance. Similar benefits have been reported in Parkinson’s disease, where it improves motor symptoms such as rigidity, tremor, and bradykinesia [12,13,14,15,16].

In the context of glaucoma, citicoline demonstrates significant neuroprotective potential. In vitro models show reduced apoptosis and synaptic loss in retinal neurons exposed to high glucose or glutamate following citicoline treatment. Animal studies also revealed anti-apoptotic effects on RGCs, preservation of retinal architecture, and protection against glutamate-induced excitotoxicity [17,18,19].

Clinically, intramuscular, oral, and topical citicoline are associated with improved visual function in patients with glaucoma. VF testing, pattern electroretinography (PERG), and visual evoked potential (VEP) demonstrate enhanced retinal and cortical responses, with long-term studies confirming sustained functional improvement. Oral formulations have demonstrated comparable efficacy to parenteral administration, and topical eye drops have shown bioavailability within the vitreous, accompanied by improvements in RGCs function and VF indices, independent of the reduction in IOP [20,21,22].

Randomized trials further validated the efficacy of topical citicoline, particularly in patients with advanced baseline dysfunction. Citicoline is now authorized in the European Union as a novel food ingredient, permitting its inclusion in food supplements and dietary foods for special medical purposes. In Italy, the Ministry of Health has recognized citicoline as a dietary supplement specifically indicated for glaucoma patients who continue to experience progressive VF loss despite achieving adequate IOP control with pharmacological therapy [11,23,24,25].

However, evidence is limited by variability in dosage regimens across studies and the frequent absence of structural endpoints, making it difficult to draw firm conclusions about long-term efficacy. The short-term effects of oral citicoline on retinal structural parameters in patients with POAG have recently been studied. Fifty-four patients were divided into two groups: 27 received 250 mg/day of oral citicoline in addition to standard topical hypotensive therapy, while 27 served as controls. Optical coherence tomography (OCT) measurements were taken at baseline, after 3 months of treatment, and again following a 1-month washout period in the citicoline group. The results showed a significant increase in average retinal nerve fiber layer (RNFL) thickness at 3 months in the citicoline group, with partial regression after discontinuation. In particular, increases in RNFL thickness in the average and inferior quadrants were significantly greater than in the controls, while no significant changes were observed in the macular ganglion cell–inner plexiform layer (mGCIPL) or other RNFL quadrants. These findings suggest that oral citicoline may exert short-term neuroprotective effects by stabilizing RNFL thickness in POAG patients, potentially slowing glaucomatous progression [26].

Recently, a multicenter, randomized, double-masked, placebo-controlled crossover trial was conducted to evaluate the impact of citicoline oral solution (500 mg/day) on vision-related quality of life in POAG patients. Quality of life was assessed using the National Eye Institute Visual Function Questionnaire-25 (VFQ-25) and the SF-36 at baseline and 3-, 6-, and 9-month follow-up visits. This trial demonstrates that treatment with citicoline can positively influence patient-reported quality of life outcomes in glaucoma compared to placebo [27].

Although a recent systematic review does not demonstrate a significant impact of citicoline on IOP reduction, RGC preservation, VF improvement, or retinal function in glaucoma patients, emerging evidence suggests that citicoline may serve as a promising adjunctive therapy. Its favorable safety profile and growing support for neuroprotective properties, independent of IOP-lowering mechanisms, underscore the need for further randomized, prospective studies to determine optimal dosing and treatment duration [11,28] (Table 1).

### 2.2. Coenzyme Q10

CoQ10, a lipophilic benzoquinone, is a vital cofactor in the mitochondrial electron transport chain, where it facilitates ATP production by shuttling electrons from complexes I and II to complex III. In addition to its bioenergetic function, CoQ10 exerts potent antioxidant activity by scavenging reactive oxygen species (ROS) and protecting lipids, proteins, and deoxyribonucleic acid (DNA) from oxidative damage. Mitochondrial dysfunction and oxidative stress are increasingly recognized as central mechanisms in the pathogenesis of glaucomatous neurodegeneration, implicating CoQ10 as a potential therapeutic candidate [29].

Preclinical studies have consistently demonstrated the neuroprotective efficacy of CoQ10 in models of retinal ischemia and excitotoxic injury. Intraocular administration of CoQ10 significantly mitigates the death of RGCs by attenuating glutamate accumulation, preserving mitochondrial membrane potential, and preventing activation of the mitochondrial permeability transition pore (PTP). These effects are associated with reduced cytochrome c release and inhibition of downstream caspase-9 and caspase-3 activation. This suggests that CoQ10 directly modulates the intrinsic apoptotic pathway [4].

Topical application of CoQ10, particularly in combination with vitamin E (Vit.E), has been shown to improve its ocular bioavailability and therapeutic efficacy. In rodent models of ocular hypertension and staurosporine-induced apoptosis of RGCs, CoQ10-containing eye drops reduced RGCs loss and apoptotic markers, including Bax, while increasing anti-apoptotic signals such as pBad. CoQ10 was also found to suppress the overactivation of NMDA glutamate receptors and the associated accumulation of nitric oxide, further implicating it in the inhibition of glutamate-mediated excitotoxicity [30,31,32,33]. The neuroprotective role of topical CoQ10 + Vit.E was further demonstrated in a recent study involving 12 rats assigned to either a sham-treated glaucoma model or a CoQ10 + Vit.E treatment group, with therapy applied for 4 weeks. Immunohistochemistry (Brn-3a, glial fibrillary acid protein (GFAP), and ionized calcium-binding adapter molecule 1) and Western blot analysis (GFAP, Bax, Bcl-xL, and transcription factor A (Tfam)) were performed. CoQ10 + Vit.E significantly preserved Brn-3a-positive RGCs (22.2 ± 4.8 vs. 15.0 ± 1.0, *p* < 0.05) and reduced GFAP-positive astroglial counts (2.5 ± 1.5 vs. 11.7 ± 2.1, *p* < 0.05) compared to the sham. The treatment also decreased Iba1 expression, upregulated Bcl-xL, and maintained Tfam levels, indicating reduced apoptosis and preserved mitochondrial function [34].

Furthermore, CoQ10 modulates glial responses in the glaucomatous retina. It inhibits oxidative stress-induced activation of ONH astrocytes and downregulates stress response proteins such as superoxide dismutase 2 (SOD2) and heme oxygenase-1 (HO-1). In ischemia–reperfusion models, CoQ10 preserves mitochondrial biogenesis by maintaining the expression of mitochondrial Tfam. It also prevents the loss of mitochondrial DNA, supporting its role in sustaining mitochondrial function under pathological conditions [33].

In a prospective, randomized clinical study, Ozates et al. [35] evaluated the effect of topical CoQ10 + Vit.E on oxidative stress markers in eyes with pseudo-exfoliative glaucoma (PXG) compared with untreated PXG and pseudo-exfoliation syndrome (PXS). Sixty-four eyes from 64 patients undergoing phacoemulsification with intraocular lens implantation were included. Aqueous humor samples were collected at the start of surgery to measure superoxide dismutase (SOD) and malondialdehyde (MDA) levels. PXG patients receiving treatment for one month preoperatively showed significantly lower aqueous humor SOD levels compared to untreated PXG cases, while SOD levels in PXS were significantly lower than in both PXG groups. No significant differences were observed in MDA levels among the groups. These findings suggest that short-term topical CoQ10 + Vit.E administration may modulate oxidative stress in PXG by reducing SOD activity, while lipid peroxidation, reflected by MDA, remains unaffected over a one-month treatment period.

The translational relevance of these findings has been supported by clinical data. In a study by Parisi et al. [36], POAG patients receiving topical CoQ10 with D-α-tocopheryl polyethylene glycol succinate (vitamin E TPGS) showed significant improvement in retinal function, as evaluated by PERG and VEP. Specifically, patients receiving adjunctive CoQ10 therapy demonstrated shortened implicit times and increased response amplitudes, indicative of improved inner retinal and visual pathway function. These improvements were observed after 6–12 months of treatment, suggesting a sustained benefit. However, PERG and VEP improvements represent functional surrogate markers and may not directly predict long-term vision preservation.

In a prospective study, the neuroprotective effects of CoQ10 + Vit.E were investigated in 96 eyes from 48 POAG patients, assigned to a study group receiving CoQ10 + Vit.E in addition to timolol + dorzolamide, or a control group receiving only timolol + dorzolamide. VEP, VF parameters, and OCT measurements of the ganglion cell layer (GCL) and RNFL were assessed at baseline, 6 months, and 12 months. After 12 months, both groups showed significant thinning of the GCL and RNFL; however, the reduction in the GCL was greater in the controls. In the controls, VEP P100 implicit times increased and amplitudes decreased, whereas in the study group, P100 implicit times decreased and amplitudes increased significantly. VF preservation was observed in 67% of study eyes compared with significant mean deviation worsening in 50% of controls. These findings suggest that adjunctive CoQ10 + Vit.E therapy in POAG may help preserve inner retinal structure, enhance cortical visual responses, and maintain VF function [37].

In the DBA/2J mouse model of chronic glaucoma, dietary CoQ10 supplementation significantly enhanced RGCs’ survival and preserved axonal integrity in the optic nerve head. These effects were accompanied by reduced expression of GFAP, indicating suppression of astroglial activation, and downregulation of the NMDA receptor subunits NR1 and NR2A. Furthermore, CoQ10 reduced apoptotic signaling and preserved components of the oxidative phosphorylation (OXPHOS) machinery, highlighting its comprehensive protective action against mitochondrial stress and neurodegeneration [38].

Dietary supplementation with ubiquinol also promoted RGCs’ survival and inhibited apoptosis in a mouse model of retinal ischemia induced by acute IOP elevation. Ubiquinol (1%) significantly increased RGCs’ survival at 2 weeks and suppressed astroglial and microglial activation. Mechanistically, ubiquinol decreased active Bax expression, preserved Bad phosphorylation (Ser112) and Bcl-xL levels at 12 h, and prevented caspase-3-mediated apoptotic cell death. These findings indicate that ubiquinol enhances RGCs’ survival by modulating the Bax/Bad/Bcl-xL apoptotic pathway, suggesting therapeutic potential for ischemic retinal degeneration associated with elevated IOP [39,40].

Due to limited dietary bioavailability, supplementation is often necessary to achieve effective plasma concentrations. A recent bioavailability study evaluated a novel extended-release oral formulation of CoQ10 with Miniactives^®^ technology in healthy adults. In the single-dose phase, the peak concentration (Cmax) occurred at 4 h, with mean values of AUCt and Tmax of 8754 μg/mL h and 4.29 h, respectively. In the multiple-dose phase, plasma levels increased within 7 days of starting therapy and remained high throughout the administration period. These results suggest that Miniactives^®^ technology enables sustained high CoQ10 blood concentrations without sharp declines [41].

Together, the body of evidence underscores the therapeutic potential of CoQ10 in glaucoma. Its multifaceted neuroprotective effects, ranging from antioxidant activity and mitochondrial preservation to the inhibition of excitotoxic and apoptotic signaling, position CoQ10 as a promising adjunct to conventional IOP-lowering strategies. Future clinical trials are warranted to confirm its efficacy in slowing glaucoma progression and preserving visual function, particularly in patients who exhibit evidence of mitochondrial dysfunction or damage related to oxidative stress [42] (Table 2).

### 2.3. Pyruvate

Oxidative stress and metabolic dysfunction are major pathogenic factors that contribute to neurological disorders. Pyruvate has emerged as a promising therapeutic agent for correcting neuronal network abnormalities associated with these conditions [43]. Pyruvate plays a multifaceted role in glaucoma, particularly in supporting RGC health through its involvement in energy metabolism and neuroprotection [44]. Research indicates that pyruvate and related metabolites can modulate resilience against glaucoma by influencing metabolic pathways. Collectively, these findings highlight the protective role of pyruvate-related metabolism against glaucoma and suggest potential avenues for therapeutic intervention [45].

Its therapeutic potential is supported by several mechanisms. First, oxidative stress, a hallmark of many neurological diseases, leads to the accumulation of ROS. Pyruvate serves as a potent ROS scavenger and can significantly enhance the antioxidant defense system under pathological conditions. Second, oxidative stress-induced overactivation of poly(ADP-ribose) polymerase-1 (PARP-1) depletes cytosolic NAD^+^, inhibits glycolysis, and contributes to energy failure and cell death. Pyruvate mitigates PARP-1 overactivation and, as a direct mitochondrial substrate, supports energy production independently of cytoplasmic redox status. This allows it to bypass the metabolic restrictions imposed by PARP-1. Third, pyruvate reduces blood glutamate levels and promotes clearance from brain tissue via the blood–brain barrier, thereby alleviating glutamate-induced excitotoxicity. Fourth, by increasing glycogen reserves, pyruvate improves neuronal tolerance to ischemic and hypoglycemic conditions. Fifth, it exerts potent anti-inflammatory effects, relevant for many neuroinflammatory disorders. Finally, pyruvate helps prevent neural network hyperexcitability. In summary, in addition to its well-established role in energy metabolism, pyruvate demonstrates significant neuroprotective properties, thus suggesting that it has considerable but underrecognized therapeutic potential in the treatment of neurological disorders, including Parkinson’s disease and ischemic brain injury [43,46,47].

Oxidative stress plays a key role in the pathophysiology of glaucoma, and the antioxidant properties of ethyl pyruvate may contribute to its protective effects [46]. Ethyl pyruvate, a derivative of pyruvate, is a potent antioxidant that is well tolerated by human trabecular meshwork cells [48]. In an ex vivo cultured retina model, pyruvate attenuated oxidative stress. It exhibits dual functionality, acting as both a ROS scavenger and a metabolic agonist. Through its antioxidant capacity, pyruvate is anticipated to mitigate the oxidative degradation of polyunsaturated fatty acids, which are essential for preserving the structural and functional integrity of neural tissues [49]. Pyruvate also exhibits neuroprotective effects on RGCs, including under conditions of glucose deprivation in cell culture models. In this model, oral supplementation of the glycolytic product pyruvate strongly protected against neurodegeneration in both rat and mouse glaucoma models [50].

Since mitochondria rely on lactate or glycolysis-derived pyruvate to generate ATP, a key question is whether energy substrate availability or mitochondrial defect contributes to the energy compromise observed before axon degeneration in glaucoma. This hypothesis was prompted by the observation of markedly reduced expression of glucose and monocarboxylate transporters in the optic nerve of D2 mice, human optic nerve samples, and in mice subjected to acute glaucomatous injury.

To evaluate this, Harun-or-Rashid et al. [51] placed D2 mice and control mice on a ketogenic diet to promote mitochondrial function. After eight weeks, the diet enhanced mitochondrial biogenesis, restored monocarboxylate transporter expression, improved energy availability, reduced glial hypertrophy, preserved RGCs and their axons, and maintained normal physiological signaling to central visual targets. This neuroprotective effect was also accompanied by a robust antioxidant response. Overall, these findings suggest that energy failure and subsequent axonal degeneration in D2 mice are driven primarily by reduced substrate availability, secondary to the downregulation of the key metabolic transporter.

**Figure 1:**
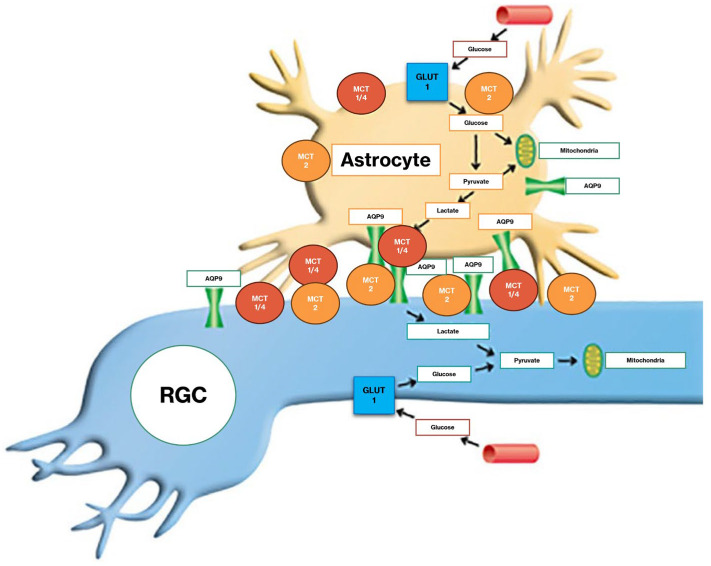
Representation of the energy transportation between astrocytes and retinal ganglion cells (RGCs). Aquaporin 9 (AQP9) and monocarboxylate transporters (MCTs) along with lactate and pyruvate transport from astrocyte to RGCs [54].

Monocarboxylate transporter 1 (MCT1) is predominantly expressed in glial cells, consistent with a role in lactate and pyruvate export, whereas MCT2 is localized to neurons, including RGCs, indicating an import-oriented function [52,53]. MCT1, MCT2, and MCT4 may at least partly interact with AQP9 in the retina. Evidence is accumulating that AQP9 functions through protein–protein interactions (Figure 1) [54].

Preclinical studies have demonstrated that oral supplementation with pyruvate protects against neurodegeneration in glaucoma models. In both rat and mouse models, pyruvate supplementation preserved RGCs and optic nerve fibers, supporting its potential as a neuroprotective agent in glaucoma [50]. Notably, overexpression of MCT2 has been shown to confer neuroprotection to RGCs in both DBA/2J mice and in the bead-induced ocular hypertension model. This overexpression, which facilitates pyruvate transport, also improves energy homeostasis in the glaucomatous visual system. This finding suggests that enhancing pyruvate availability to RGCs may be a viable strategy to combat neurodegeneration in glaucoma [53]. Conversely, pharmacological inhibition of MCTs abolishes the neuroprotective effects of pyruvate on RGCs in vitro [50].

Recent findings have demonstrated a progressive decline in retinal pyruvate levels in DBA/2J mice in response to elevated IOP. This decline was accompanied by transcriptional dysregulation of genes involved in glycolysis and pyruvate metabolism [50]. Long-term oral supplementation with pyruvate significantly mitigated optic nerve degeneration, preserved axonal transport, counteracted IOP-induced metabolic disturbances, and improved visual function of RGCs [44].

In summary, pyruvate contributes to glaucoma management by supporting mitochondrial function, enhancing energy metabolism, providing antioxidant protection, and potentially improving visual function. However, the majority of evidence comes from preclinical studies (animal and cellular models) and from trials of combination metabolic therapies (e.g., nicotinamide plus pyruvate). There are currently no published clinical trials demonstrating the efficacy of pyruvate monotherapy for glaucoma in humans. Thus, clinical translation remains unproven and requires further investigation [55] (Table 3).

### 2.4. Nicotinamide

Multiple pathways, both directly and indirectly linked to pathological alterations in mitochondrial metabolism, have been implicated in mitochondrial dysfunction and neuronal cell death. Among these, disruptions in nicotinamide adenine dinucleotide (NAD^+^) metabolism play a pivotal role in neurodegenerative processes. NAD^+^ and its reduced form, NADH, function as essential cofactors in redox reactions and cellular signaling in all forms of life. They are integral to key metabolic pathways, including glycolysis, the tricarboxylic acid (TCA) cycle, and oxidative phosphorylation (OXPHOS). These processes converge to generate adenosine triphosphate (ATP), the universal energy currency of cells. Consequently, NAD^+^/NADH redox balance is critical for ATP production, which is necessary for the propagation of action potentials along RGCs axons. Furthermore, NAD^+^ serves as a substrate for NAD^+^-consuming enzymes such as sirtuins, poly (ADP-ribose) polymerases (PARPs), and cyclic ADP-ribose synthases (cADPRs; including CD38 and CD157). These enzymes regulate essential cellular functions, including signal transduction, DNA repair, cell proliferation, aging, and epigenetic regulation [56].

Nicotinamide riboside (NR), a naturally occurring NAD^+^ precursor and vitamin B3 derivative, enhances NAD^+^ biosynthesis through the NRK1/2 and NMNAT pathways and has shown efficacy in improving mitochondrial function in animal models. However, data on its pharmacokinetics and NAD^+^-boosting potential in humans remain limited [57].

Airhart et al. [57] found that oral NR supplementation was well tolerated, with no adverse events. Significant increases in both NR and NAD^+^ concentrations occurred from baseline to steady state on Day 9, with NAD^+^ levels doubling. A strong positive correlation was noted between changes in NR and NAD^+^ levels. Routine laboratory biomarkers remained within normal limits, supporting the safety profile of NR. These findings demonstrate that NR effectively elevates circulating NAD^+^ levels in healthy individuals, supporting its potential as a therapeutic agent for conditions characterized by mitochondrial dysfunction.

Williams et al. [58,59] investigated the early molecular mechanisms underlying glaucomatous neurodegeneration and evaluated potential metabolic interventions. Using RNA sequencing and unsupervised hierarchical clustering in a mouse model of IOP elevation, they identified early transcriptomic alterations in RGCs, including upregulation of genes linked to mitochondrial dysfunction and oxidative phosphorylation. These changes occurred before overt neurodegeneration. Electron microscopy confirmed mitochondrial structural abnormalities, and biochemical assays revealed decreased NAD^+^/NADH and glutathione levels. NAD^+^ depletion, observed in both aging and glaucoma-prone mice, was hypothesized to impair neuronal energy metabolism and increase susceptibility to stress, contributing to RGC loss. To test this, the authors supplemented glaucoma model mice with nicotinamide, an NAD^+^ precursor. At physiologically relevant doses, nicotinamide prevented RGC degeneration and functional decline in a dose-dependent manner without adverse effects. Additionally, intravitreal gene therapy promoting NAD^+^ biosynthesis provided substantial neuroprotection, and the combination of nicotinamide plus gene therapy produced the most robust protection. These results highlight early mitochondrial and metabolic dysfunction as contributors to glaucomatous damage, suggesting that NAD^+^ augmentation may be a viable neuroprotective strategy [60].

In a crossover, double-masked, randomized clinical trial involving 57 participants with diagnosed and treated glaucoma from two tertiary care centers, nicotinamide supplementation on inner retinal function was evaluated in individuals receiving concurrent glaucoma therapy. Participants received oral placebo or nicotinamide, starting with 1.5 g/day for 6 weeks followed by 3.0 g/day for another 6 weeks, and then crossed over to the alternate treatment without a washout period. Inner retinal function was assessed using photopic negative response (PhNR) parameters via electroretinography and perimetry. Nicotinamide significantly improved PhNR saturated amplitude (Vmax) by 14.8%, compared with a non-significant 5.2% improvement on placebo. The Vmax ratio increased by 12.6% with nicotinamide versus 3.6% on placebo. Twenty-three percent of participants showed PhNR Vmax improvement beyond the 95% coefficient of repeatability on nicotinamide compared to nine percent on placebo. A trend toward improved VF mean deviation was observed, with 27% of participants improving by ≥1 dB on nicotinamide and fewer showing deterioration (4%) compared to placebo. While these findings suggest that nicotinamide may enhance inner retinal function in glaucoma patients, the trial was relatively short-term (12 weeks per treatment arm) and did not evaluate long-term structural preservation over years. Further research is therefore warranted to determine whether these functional benefits translate into sustained neuroprotection [61].

In an ongoing multicenter, randomized, double-blind, placebo-controlled, parallel-group trial involving 125 POAG patients, Leung et al. [62] are evaluating whether NR can slow optic nerve degeneration. Participants receive 300 mg of NR or placebo daily for 24 months. Assessments include clinical exams, RNFL evaluation, and VF testing at baseline, 1 month, 4 months, and every 2 months until study completion. Traditional neuroprotection trials rely on VF progression, which is variable and takes time to confirm. This study instead uses RNFL thinning as a primary outcome, potentially enabling detection of effects in a shorter timeframe. It may also establish a practical model for future glaucoma neuroprotection trials.

In addition, Williams et al. [58] reported that oral nicotinamide and/or gene therapy (driving Nmnat1 expression) protected against glaucoma both prophylactically and as an intervention. At the highest dose, 93% of eyes did not develop glaucoma. This supports therapeutic use of vitamin B_3_ in glaucoma and potentially other age-related neurodegenerations.

Although niacin is a form of vitamin B3 and nicotinamide is its amide derivative, the American Glaucoma Society and American Academy of Ophthalmology do not consider them interchangeable. Currently, niacin is not being tested in human glaucoma trials, and its hepatotoxicity risk is dose-dependent, particularly at therapeutic doses. In contrast, nicotinamide is generally well tolerated at clinical trial doses and has a more favorable safety profile for long-term use [63]. Clinical studies should determine optimal dosing and safety in humans. Advances in trial design now allow neuroprotective agents to be evaluated in smaller, shorter-duration cohorts, particularly in high-risk patients. These findings provide compelling preclinical evidence that early metabolic intervention may delay or prevent glaucomatous neurodegeneration, highlighting nicotinamide as a promising candidate for therapeutic translation (Table 4).

### 2.5. Pyrroloquinoline Quinone

A newly identified class of redox-active enzymes, known as quinoenzymes, use ortho-quinones derived from either tyrosine or tryptophan as coenzymes or cofactors. PQQ was the first ortho-quinone cofactor characterized. As reported in earlier studies, PQQ has high solubility, remarkable thermal stability, and the ability to undergo sustained redox cycling. Chemically, PQQ integrates several advantageous properties found in other cofactors: the reducing potential of ascorbic acid, the redox capabilities of riboflavin, and the carbonyl reactivity of pyridoxal. On a molar basis, PQQ shows redox cycling efficiency that is at least two orders of magnitude greater than that of ascorbic acid, menadione, and all tested isoflavonoids and polyphenolic compounds [55].

PQQ has been shown to influence energy metabolism and neurological function in animal models, primarily through the modulation of cellular signaling pathways and mitochondrial activity. However, the physiological effects of PQQ in humans remain insufficiently defined. To address this gap, a crossover study was conducted involving ten healthy adult volunteers (five females and five males) who received PQQ in a beverage under two experimental conditions (Study 1 and Study 2).

In Study 1, participants received a single oral dose of PQQ at 0.2 mg/kg body weight. Serial measurements of plasma and urinary PQQ concentrations were performed alongside evaluations of antioxidant status over a 48 h period using total peroxyl radical-trapping potential and thiobarbituric acid-reactive substance (TBARS) assays. In Study 2, participants were given a daily dose of 0.3 mg/kg PQQ for a total duration of 76 h. Post-intervention analyses included assessments of systemic inflammatory markers, specifically plasma C-reactive protein (CRP) and interleukin-6 (IL-6), in addition to routine clinical biomarkers (e.g., total cholesterol, glucose, HDL, LDL, and triglycerides). Furthermore, urinary metabolomic profiling was conducted using proton nuclear magnetic resonance (1H-NMR) spectroscopy, with particular emphasis on metabolites indicative of oxidative metabolism. However, the human sample size was small (n = 10), which limits the generalizability of these findings [64].

Routine clinical parameters remained within established reference ranges and were not significantly altered by PQQ supplementation. In contrast, acute PQQ administration in Study 1 was linked to an apparent enhancement of antioxidant capacity, as evidenced by a reduction in TBARS levels. In Study 2, sustained PQQ supplementation led to statistically significant reductions in systemic inflammatory biomarkers, including CRP and IL-6, as well as decreased urinary excretion of methylated amines such as trimethylamine N-oxide. Moreover, metabolomic profiling revealed urinary alterations consistent with improved mitochondrial function.

Collectively, these findings represent some of the first human data to parallel the systemic biological effects of PQQ previously observed in animal models, supporting its potential role in modulating oxidative metabolism and inflammatory processes in humans [64] (Table 5).

### 2.6. Homotaurine

Homotaurine (3-amino-1-propane sulfonic acid, tramiprosate) is a natural aminosulfonate compound endowed with neuromodulatory effects [65] and multiple neuroprotective properties, including the ability to counteract oxidative DNA damage. It has antifibrillogenic, antinociceptive, and analgesic activities, functioning as both a neuroprotective and neurotrophic agent. Additionally, homotaurine inhibits the formation of β-amyloid plaques, which are implicated in neuronal apoptosis and in the pathogenesis of various neurodegenerative disorders within the central nervous system [66,67].

Homotaurine also exerts a direct effect on neuronal activity. Through its affinity with GABA A receptors, it modulates cortical inhibitory activity by decreasing the neuronal response to glutamate-induced excitatory stimuli [68,69]. Emerging evidence suggests its potential relevance in the ophthalmic domain [70] (Table 6).

### 2.7. Berberine

Berberine (BRB), a naturally occurring isoquinoline alkaloid, has a broad spectrum of pharmacological activities. In recent years, considerable research has focused on elucidating its role in central nervous system disorders. Notably, its potential therapeutic effects in neurodegenerative diseases, such as Alzheimer’s disease, Parkinson’s disease, and Huntington’s disease, have garnered significant attention. Emerging evidence suggests that BRB may attenuate neuroinflammation, oxidative stress, and endoplasmic reticulum stress, thereby mitigating neuronal injury and apoptosis. Despite these promising findings, the precise molecular mechanisms underlying its neuroprotective actions are not yet fully understood and require further investigation [71] (Table 7).

Campisi et al. [72] studied the effects of BRB and an alkaloid-rich extract derived from the roots of *B. aetnensis* on glutamate-induced upregulation of tissue transglutaminase (TG2) in primary rat astrocyte cultures, used as an in vitro model of excitotoxicity. Analytical findings confirmed that BRB was the principal constituent of the root alkaloid extract. Both BRB and the alkaloid extract effectively restored glutamate-induced alterations in oxidative stress markers and normalized TG2 expression levels. Moreover, treatment with BRB or the extract attenuated key pathological features of excitotoxicity, including excessive glutamate production, protein misfolding and aggregation, mitochondrial fragmentation, and neurodegenerative changes. These results indicate that berberine and the alkaloid extract of *B. aetnensis* roots may hold promise as natural therapeutic agents for the treatment of excitotoxicity-associated neuropathologies.

BRB preserved the thickness of the outer nuclear layer (ONL) and maintained RGCs’ integrity following light-induced retinal damage. BBR significantly attenuated photoreceptor apoptosis, suppressed the expression of the pro-apoptotic marker c-fos, and reduced the pro-inflammatory activation of Müller glial cells, along with the expression of inflammatory cytokines such as TNF-α and IL-1β. Additionally, BBR treatment led to a downregulation of P2X7 receptor (P2X7R) protein levels. Double immunofluorescence analysis demonstrated that BBR decreased P2X7R overexpression in both RGCs and Müller cells. Notably, co-administration of BBR with the P2X7R agonist BzATP eliminated the protective effects of BBR on retinal structure and photoreceptor survival. In contrast, BBR treatment in P2X7R knockout (KO) mice produced an additive protective effect, increasing ONL thickness and raising the number of photoreceptors. These findings support the view that the P2X7 receptor plays a key role in light-induced retinal damage and that BBR mitigates this damage by limiting histological degeneration, cellular apoptosis, and inflammatory responses [73].

Berberine also appears to be a promising preventive or adjuvant treatment for diabetic retinopathy, and its key protective effect may involve the regulation of RGCs apoptosis through the GABA-alpha receptor/protein kinase C-alpha (GABAAR/PKC-α) pathway [74].

However, BRB’s oral bioavailability is limited. This is primarily due to extensive intestinal first-pass metabolism by cytochrome P450 enzymes (CYPs), poor absorption related to low solubility, P-glycoprotein (P-gp)-mediated efflux transport, and hepatic first-pass metabolism in rats. Various formulation strategies aim to enhance the oral bioavailability of BBR by mitigating these limiting factors. Additionally, exploring alternative administration routes that bypass first-pass metabolism could further improve BBR’s systemic availability [75].

### 2.8. Gamma-Aminobutyric Acid

Glutamate and gamma-aminobutyric acid (GABA) are the primary excitatory and inhibitory neurotransmitters, respectively, within the retina. Glutamate acts as the principal neurotransmitter at synapses between photoreceptors and bipolar cells, as well as between bipolar cells and RGCs. Conversely, GABA is used predominantly by horizontal and amacrine cells within the lateral pathways, where it modulates synaptic transmission across the outer and inner plexiform layers. Several studies have implicated glutamatergic signaling in the pathophysiology of glaucoma, but the role of GABAergic mechanisms remains comparatively underexplored. A homeostatic balance between excitatory glutamatergic and inhibitory GABAergic activity is thought to be critical for preserving retinal function and accurately encoding visual information. Disruption of this balance may contribute to retinal dysfunction and neurodegeneration. Significant impairment of the GABAergic system has been observed in experimental models of elevated IOP, such as rat retinas treated with hyaluronic acid.

Bailey et al. [76] found that the butanoate metabolism pathway overall, and specifically the aspects of the pathway that contribute to GABA and acetyl-CoA metabolism, was the only pathway significantly associated with both POAG and NPG. Collectively these results implicate GABA and acetyl-CoA metabolism in glaucoma pathogenesis and suggest potential therapeutic targets.

Using proton magnetic resonance spectroscopy and functional magnetic resonance imaging, Bang et al. [77] investigated the GABAergic and glutamatergic systems in the visual cortex of individuals with glaucoma. Neural specificity, a functional property influenced by GABA and glutamate signaling and essential for efficient sensory and cognitive processing, was also examined. They reported that, in older adults, both GABA and glutamate levels declined with increasing severity of glaucoma regardless of chronological age. In particular, only the reduction in GABA levels was significantly associated with a decrease in neural specificity. This relationship remained significant after controlling for retinal structural damage, age, and visual cortex gray matter volume. These findings suggest that a glaucoma-specific decline in GABA may contribute to impaired neural specificity in the visual cortex, highlighting the potential of targeting GABAergic pathways to enhance neural processing in glaucoma.

Furthermore, Zhou et al. reported that the activation of 5-HT1A receptors in retinas facilitated presynaptic GABA release functions by suppressing cAMP-PKA signaling and decreasing PKA phosphorylation, which could lead to the de-excitation of RGC circuits and suppress excitotoxic processes in glaucoma [78].

Therapeutic strategies targeting GABA receptors or improving GABAergic activity may offer neuroprotective benefits by reducing excitotoxicity and promoting neuronal survival. Although research is still evolving, modulating GABA pathways represents a promising adjunctive approach to current glaucoma treatments, potentially helping to preserve vision and slow disease progression beyond conventional intraocular pressure-lowering therapies (Table 8).

## 3. Promising Neuroprotective Molecules

Novel neuroprotective molecules in glaucoma offer promising strategies to preserve RGCs and prevent vision loss progression. Several pharmacological and natural compounds have been investigated for their neuroprotective and cytoprotective potential in glaucoma (Table 9).

Neurosteroids are endogenous steroids synthesized in the central and peripheral nervous systems that play a vital role in modulating neuronal excitability, neuroprotection, and synaptic function. In recent years, their potential therapeutic role in ocular diseases, including glaucoma, has garnered increasing attention. Emerging evidence suggests that neurosteroids may help protect retinal ganglion cells through anti-inflammatory, anti-apoptotic, and neuroprotective mechanisms.

Allopregnanolone is a neurosteroid and a powerful modulator of neuronal excitability. The neuroprotective effects of allopregnanolone include potentiation of GABA inhibitory responses. Pressure loading at 75 mmHg was found to significantly increase allopregnanolone levels as measured by liquid chromatography and tandem mass spectrometry and by immunochemistry. Elevated hydrostatic pressure also increased neurosteroid immunofluorescence, especially in the GCL and inner nuclear layers, while staining was negligible at lower pressures. Enhanced allopregnanolone levels and immunostaining were substantially blocked by finasteride but were more effectively inhibited by dutasteride and D-2-amino-5-phosphonovalerate. Administration of exogenous allopregnanolone suppressed pressure-induced axonal swelling in a concentration-dependent manner, while picrotoxin reversed these neuroprotective effects. These results indicate that the synthesis of allopregnanolone is mainly enhanced through N-methyl-D-aspartate receptors in the pressure-loaded retina and that allopregnanolone reduces pressure-mediated retinal degeneration via GABAA receptors. Allopregnanolone and other related neurosteroids may serve as potential novel therapeutic targets for the prevention of pressure-induced retinal damage in glaucoma [79].

Understanding the role of neurosteroids in glaucoma may open new avenues for targeted, neuroprotective treatment strategies beyond intraocular pressure control.

Riluzole (2-amino-6-(trifluoromethoxy) benzothiazole) is a neuroprotective agent that inhibits glutamate release by blocking voltage-dependent Na^+^ channels. Owing to this feature, it is used in amyotrophic lateral sclerosis and helps reduce the progression of motor neuron damage.

Yildiz et al. [80] evaluated the neuroprotective effects of riluzole via the modulation of matrix metalloproteinase-2 (MMP-2) and MMP-9 expression in an experimental glaucoma model in rats. Twenty-eight Wistar albino rats were divided into four groups: control (Group I), glaucoma (Group II), glaucoma with vehicle treatment (Group III; corn oil + DMSO), and glaucoma with riluzole treatment (Group IV; corn oil + dimethyl sulfoxide (DMSO) + riluzole). Glaucoma was induced by episcleral vein cauterization in the left eyes of Groups II–IV. Riluzole (5 mg/kg) was administered intraperitoneally to Group IV for 7 weeks. IOP rose significantly in all groups in which glaucoma was induced compared to controls (*p* < 0.001), but riluzole treatment led to a reduction in IOP relative to the untreated glaucoma group. Quantitative PCR and immunohistochemical analyses revealed a slight reduction in MMP-2 and MMP-9 expression in riluzole-treated eyes. Histopathological assessments did not reveal substantial differences in RGCs degeneration, hemorrhage, or layer differentiation across the glaucoma groups. Notably, the reduced MMP-2 and MMP-9 expression observed in both the riluzole- and vehicle-treated groups suggests a potential influence of the solvent (corn oil + DMSO), necessitating further investigation to clarify riluzole’s specific contribution.

Green tea is another substance that has recently attracted interest in the treatment of glaucoma. Currently, there is limited knowledge about how green tea influences the human body, including its effects on IOP. As the least processed form of tea, green tea retains the highest levels of antioxidants and beneficial polyphenols. Among these, catechins are a major group, with epigallocatechin gallate (EGCG) being the most plentiful, accounting for about 50% of all catechins and the most biologically active. In an experimental acute IOP elevation rat model (110 mmHg for 2 h), oral administration of green tea extract demonstrated anti-oxidative and anti-inflammatory effects on ischemia-injured RGCs. However, this IOP elevation was acute, and the potential long-term effects of green tea extract on IOP or RGCs’ survival in chronic glaucoma remain unknown [81].

A study involving 43 young volunteers examined the effects of green tea and EGCG on IOP. Participants were divided into three groups: green tea extract (17 subjects), EGCG extract (17 subjects), and placebo control (9 subjects). Each group consumed a 400 mg capsule of their assigned treatment. The results demonstrated that both the green tea and EGCG groups showed statistically significant reductions in IOP, whereas no significant changes were observed in the placebo group throughout the study period. These findings suggest that moderate consumption of green tea or EGCG extracts may help lower IOP, offering potential benefits for individuals with elevated IOP or those at risk of developing glaucoma [81].

Among acetylcholinesterase (AChE) inhibitors, donepezil, widely used in Alzheimer’s disease, has shown promising effects in retinal neurodegeneration. In vitro studies indicate that donepezil reduces mutant optineurin accumulation and stimulates autophagy, protecting RGCs from apoptosis [82]. It has also improved retinal histology in animal ischemia–reperfusion models [83]. In a prospective study of 32 newly diagnosed Alzheimer’s patients with normal IOP, a daily dose of 5 mg donepezil for four weeks reduced the mean IOP from 14.1 mmHg to 12.8 mmHg (8.8% reduction) and decreased the pupil diameter from 3.9 mm to 3.6 mm (7.4% reduction). These results show that systemic donepezil induces miosis and lowers IOP in normotensive individuals, suggesting that it could serve as an adjunctive glaucoma therapy in Alzheimer’s patients [84].

Galantamine and rivastigmine, other AChE inhibitors, exhibit similar mechanisms. Galantamine, in particular, has demonstrated the ability to increase ocular blood flow and protect against RGC death in animal models of ocular hypertension, likely through the modulation of cholinergic signaling and vascular regulation [85]. While rivastigmine lacks direct glaucoma-specific data, its cholinergic neuroprotection supports further exploration in this context.

Memantine, an NMDA receptor antagonist, was among the first neuroprotective agents to be tested extensively in glaucoma. It effectively prevented RGC loss and trans-synaptic degeneration in rodent and primate models [86]. Despite strong preclinical evidence suggesting neuroprotective effects, memantine failed in phase III glaucoma trials likely because the drug’s clinical effect was too small to detect over the study period, the patient population had slowly progressive disease, and functional endpoints such as visual field change lacked the sensitivity to capture subtle neuroprotection [87]. Nonetheless, novel memantine derivatives such as MN-08 are undergoing renewed investigation and show improved pharmacodynamics and efficacy in more recent studies [88].

Selegiline, a monoamine oxidase-B inhibitor primarily used in Parkinson’s disease, has also shown neuroprotective properties in retinal models. It prevents apoptosis by modulating the Bcl-2 pathway, promoting cell survival under oxidative stress conditions relevant to glaucoma pathogenesis [89,90].

Erythropoietin (EPO) and its non-hematopoietic derivative EPO-R76E have been identified as potent neuroprotective agents in glaucoma models. In DBA/2J mice, EPO administration preserved RGCs’ structure and function by activating antioxidant pathways such as NRF2/ARE and reducing inflammation [91]. Interestingly, elevated EPO levels have also been detected in the aqueous humor of glaucoma patients, possibly reflecting an endogenous attempt to counteract retinal stress [92].

Nimodipine, a calcium channel blocker, has been shown to have benefits in normal-tension glaucoma (NTG) by improving ocular perfusion, contrast sensitivity, and color vision [93]. In topical formulations combined with prostaglandin analogs like latanoprost, nimodipine also contributed to IOP reduction and enhanced ocular hemodynamics [94].

A variety of natural compounds are under investigation for their antioxidant, anti-inflammatory, and neuroprotective effects. Curcumin, a polyphenol from turmeric, has shown the ability to protect trabecular meshwork cells and RGCs from oxidative stress and may modulate the mitochondrial pathways involved in glaucomatous damage [95]. Ginseng (Panax ginseng) has been shown in clinical studies to improve visual function, reduce ocular discomfort, and enhance tear film stability in glaucoma patients, possibly via vascular and anti-inflammatory mechanisms [96]. Resveratrol, a compound found in grapes and red wine, has been reported to mitigate oxidative stress and preserve mitochondrial function in experimental glaucoma [97]. Tauroursodeoxycholic acid (TUDCA), a bile acid derivative, has demonstrated anti-apoptotic effects on RGCs in models of ocular hypertension.

Other agents such as vitamin D, creatine, and apigenin are emerging candidates in neuroprotective strategies. Vitamin D may exert anti-inflammatory effects and protect against retinal remodeling [98], while creatine supports mitochondrial energetics and may counteract bioenergetic failure in glaucomatous neurodegeneration [99]. Apigenin, a flavonoid found in chamomile and parsley, also shows promise for reducing oxidative stress in the retina.

In summary, numerous pharmacological and natural agents demonstrate significant neuroprotective potential in glaucoma through diverse mechanisms including inhibition of excitotoxicity, enhancement of mitochondrial function, reduction in oxidative stress, and promotion of ocular blood flow. While preclinical data are encouraging, translation to clinical application remains limited. Larger, well-designed clinical trials are essential to validate their efficacy and safety in glaucoma patients.

## 4. Combining Neuroprotectants: Rationale

The recent literature shows a growing interest in neuroprotective therapies aimed at preserving the structure and function of RGCs independent of IOP. Among emerging strategies, the combination of multiple neuroprotective molecules represents a promising approach. This strategy addresses the multifactorial nature of glaucomatous damage. Glaucoma pathogenesis involves a complex interaction of oxidative stress, excitotoxicity, mitochondrial dysfunction, neuroinflammation, and impaired axonal transport. Targeting a single pathogenic mechanism is unlikely to provide sufficient protection. Therefore, combining agents with complementary mechanisms of action may offer a synergistic therapeutic benefit. Another compelling rationale for combination therapy lies in the variability of patient responses to individual agents. A multi-targeted approach may enhance efficacy across a broader patient population. Furthermore, some compounds may exert additive or potentiating effects when used together, leading to greater neuroprotection than each agent could achieve alone. Importantly, the combination of neuroprotective agents also presents challenges. These include potential drug interactions, cumulative toxicity, and difficulties in optimizing dosage and delivery. Preliminary in vivo and clinical studies suggest that combination therapy may slow glaucoma progression more effectively than monotherapy, though large-scale randomized clinical trials are still lacking [100]. Ultimately, the strategic use of multiple neuroprotective agents, guided by an understanding of glaucoma’s molecular complexity, holds substantial potential for transforming the management of this blinding disease. As research progresses, identifying the most effective combinations and delivery methods will be critical to unlocking the full therapeutic value of neuroprotection in glaucoma.

Below, we present some of the most recent research supporting the combined use of neuroprotective molecules currently used for the treatment of glaucoma (Table 10).

## 5. Citicoline, Coenzyme Q10, and Vitamin B3

Among the candidate molecules for neuroprotection, citicoline and CoQ10 have emerged as particularly promising compounds due to their complementary mechanisms of action targeting mitochondrial dysfunction, oxidative stress, neurotransmitter imbalance, and apoptotic pathways [110] (Table 10).

Based on the recent literature, the fixed combination of neuroprotective agents—particularly citicoline and CoQ10, with or without the addition of vitamin B3 (niacin)—shows significant potential in the management of glaucomatous neurodegeneration and other oxidative stress-related conditions.

Both citicoline and CoQ10 have been extensively reviewed for their neuroprotective capabilities in glaucoma. These compounds exert their effects through distinct molecular pathways involved in glaucomatous neurodegeneration.

In experimental models simulating glaucomatous neurodegeneration, citicoline and CoQ10 have shown significant neuroprotective effects through complementary mechanisms. In a model of partial ON crush, progressive RGC loss was induced via a pro-apoptotic environment. Citicoline administration enhanced RGCs’ survival by upregulating anti-apoptotic proteins such as Bcl-2 and mimicking brain-derived neurotrophic factor (BDNF) activity. Similarly, CoQ10 increased Bcl-xL expression, preventing mitochondrial permeability transition and apoptosis by stabilizing calcium homeostasis, regulating p53 activity, and maintaining mitochondrial ATP synthesis and autophagy [8].

Both compounds have also been shown to counteract excitotoxicity. Citicoline reduces kainic acid-induced retinal damage by attenuating nitrosative stress and ERK1/2 activation. CoQ10 prevents glutamate-mediated excitotoxicity, preserves mitochondrial integrity, and suppresses astroglial activation in the ONH by lowering glial fibrillary acidic protein (GFAP) expression. Additionally, CoQ10 prevents the upregulation of NR1, NR2A, superoxide dismutase 2 (SOD2), and heme oxygenase 1 while modulating pro-apoptotic (Bax) and anti-apoptotic (pBad) proteins. It also maintains mitochondrial DNA and oxidative phosphorylation (OXPHOS) protein expression in glaucomatous models [8].

Furthermore, citicoline reduces active caspase-9 and -3 levels, promoting neurite regeneration, and improves mitochondrial metabolism by enhancing phospholipid synthesis and ATP production. Human studies have shown increased phosphocreatine and ATP levels, reflecting improved mitochondrial bioenergetics. In parallel, CoQ10 preserves mitochondrial structure and mitofilin expression under oxidative stress and enhances ATP production via redox cycling in mitochondria and lysosomes [8].

Citicoline and CoQ10 also exhibit anti-inflammatory effects. Citicoline reduces retinal gliosis and apoptosis, whereas CoQ10 lowers TNF-α by inhibiting NF-κB and modulating inflammatory mediators [8].

Citicoline improves cerebral blood flow by enhancing endothelial nitric oxide synthesis. Metabolically, CoQ10 supports choline oxidation and respiratory chain activity, particularly under conditions of ubiquinone depletion. Age-related declines in retinal CoQ10 levels and choline deficiency have been shown to exacerbate neurodegeneration, which CoQ10 helps counteract [8].

Altogether, the combined administration of citicoline and CoQ10 appears to exert synergistic, multi-targeted neuroprotective effects. These include enhanced mitochondrial function, reduced apoptosis and inflammation, neurotransmitter support, and improved bioenergetic balance. This combination offers a promising strategy for glaucoma management [8].

Notably, a recent in vitro study demonstrated the excellent biocompatibility of citicoline, CoQ10, and vitamin B3, both individually and in combination. Using the MTT assay on hypothalamic HypoE22 cells, the investigators found that cell viability remained consistently above 70% at the highest non-toxic concentration (10 µM) compared to untreated controls under basal conditions. This was observed across all pharmacological treatments tested. The study also showed that all three compounds individually reduced the expression of pro-inflammatory markers (IL-6 and TNFα) and enhanced brain-derived neurotrophic factor (BDNF) expression, a key mediator of neuronal survival and plasticity. However, the combination of the three agents produced the most significant effects, suggesting additive or synergistic activity. The in silico component of the study revealed predicted interactions between the compounds and key proteins involved in mitochondrial function, neurotransmitter regulation, and inflammation, supporting the observed biological outcomes [8,101].

The synergistic effects of these two molecules were further confirmed in vitro by Di Simone et al. [102], who conducted a comprehensive in vitro study using rat CTX-TNA2 astrocytes—a glial cell type involved in neuronal support and inflammation—to investigate the effects of citicoline and CoQ10, alone and in combination, on oxidative stress-induced damage. Astrocytes were exposed to hydrogen peroxide, a pro-oxidant stimulus mimicking cellular oxidative stress seen in glaucoma and other neurodegenerative conditions. Both compounds, individually and especially in combination, significantly improved cell viability, reduced apoptotic markers (e.g., BAX), increased anti-apoptotic gene expression (e.g., BCL-2), and stimulated the expression of cardiolipin synthase 1 (CRLS1), a mitochondrial enzyme essential for maintaining mitochondrial membrane stability. Additionally, they suppressed the expression of inflammatory cytokines IL-6 and TNFα and the transcription factor NFkB, which plays a central role in neuroinflammation. Importantly, the fixed combination of citicoline and CoQ10 demonstrated a greater protective effect than either compound alone, indicating a synergistic mechanism.

This synergism was further validated by the TUNEL assay, which showed a reduction in the number of apoptotic nuclei in treated astrocytes. These results support the idea that the combined administration of citicoline and CoQ10 can enhance mitochondrial protection, reduce oxidative and inflammatory stress, and promote cell survival in neural tissue. Since astrocytes are crucial in maintaining the homeostasis of the neural environment and play a role in RGC support, their protection is vital for slowing glaucomatous damage [102].

Finally, a recent study by Matamoros et al. [103] investigated the efficacy of the combined administration of citicoline and CoQ10 to modulate glial responses in a murine model of unilateral laser-induced ocular hypertension (OHT). Oral administration of citicoline and CoQ10, initiated prior to the induction of OHT and continued post-treatment, was associated with a significant reduction in IOP and a marked attenuation of both macroglial (GFAP-positive) and microglial (P2RY12-positive) activation in the retina and central visual pathways, including the dorsolateral geniculate nucleus, superior colliculus, and primary visual cortex (V1). The treatment reduced morphological signs of microglial activation, such as soma hypertrophy and decreased arbor complexity, and lowered the number of activated microglial cells. These findings indicate that citicoline and CoQ10 exert a protective effect by modulating the neuroinflammatory milieu, potentially preserving RGCs from IOP-induced degeneration.

Collectively, these studies highlight several important points. First, oxidative stress and neuroinflammation are central to glaucomatous RGC degeneration, and targeting both pathways simultaneously, offers a more robust therapeutic approach than monotherapy. Second, the combination of citicoline and CoQ10 shows strong potential in preserving both neuronal and glial cell health under stress conditions. Third, the addition of vitamin B3 may further enhance the neuroprotective effects by supporting mitochondrial energy metabolism and restoring NAD+ levels, which are often depleted in glaucomatous and aged tissues.

Furthermore, these compounds may offer a broad therapeutic window, low toxicity, and the potential for topical, oral, or injectable formulations, making them versatile tools in clinical practice. From a translational perspective, the incorporation of these agents into fixed-combination eye drops, oral supplements, or sustained-release delivery systems could provide an adjunctive strategy for glaucoma patients not fully responsive to IOP-lowering therapies. This is especially relevant for patients with normal-tension glaucoma or those with continued visual field progression despite adequate pressure control.

In conclusion, the combination of citicoline and coenzyme Q10 with or without the addition of vitamin B3 offers a scientifically grounded, multifactorial neuroprotective approach to glaucoma management. While evidence from in vitro and animal studies is compelling, further randomized, controlled clinical trials are necessary to confirm their efficacy in human populations, determine optimal dosages, and assess long-term safety. If confirmed, such combination therapies could significantly shift the paradigm of glaucoma treatment toward a neuroprotective and disease-modifying approach.

## 6. Citicoline and Vitamin B3

The rationale of combining niacin (vitamin B3) and citicoline in the diet to more effectively preserve RGCs’ function and survival than either agent alone has been investigated in a mouse model of hypertensive glaucoma by Melecchi et al. [104]. To test whether their combination produces additive or synergistic neuroprotection, the authors induced ocular hypertension in C57BL/6 J mice via a single intracameral injection of 2% methylcellulose (MCE), which clogs the trabecular meshwork and elevates IOP to ~32 mmHg for two weeks. Sixty animals were randomized into ten groups (n = 6 each): control, MCE alone, low- or high-dose niacin (0.5 g/kg/d or 2.5 g/kg/d), low- or high-dose citicoline (0.5 g/kg/d or 1 g/kg/d), and four regimens pairing each dose of niacin with each dose of citicoline. Treatments were administered in drinking water 14 days before and 14 days after the injection of MCE. IOP was monitored daily by rebound tonometry, while RGCs’ function was assessed by photopic electroretinogram (ERG; cone b-wave and photopic negative response, PhNR) and pattern ERG (PERG; N35-P50 and P50-N95 amplitudes). RGC density was quantified by RNA-binding protein with multiple splicing (RBPMS) immunohistochemistry on whole-mount retinas. Retinal levels of oxidative stress markers (nuclear factor erythroid 2-related factor 2 (Nrf-2) and Heme Oxygenase-1 (HO-1)), inflammatory mediators (phosphorylated nuclear factor kappa B (pNF-κB), IL-6, and GFAP) and intrinsic apoptotic proteins (Bax/Bcl-2 ratio, cytochrome c, and cleaved caspase-3) were measured by Western blotting [104].

MCE injection produced a sustained two-fold IOP elevation without affecting body weight or ocular transparency. Compared to the controls, untreated MCE-injected mice showed reductions in PhNR amplitude, decreases in PERG amplitudes, and lost RBPMS-positive RGCs. These functional and structural deficits were associated with the upregulation of Nrf-2 and HO-1, increases in pNF-κB and IL-6, a rise in GFAP, an elevation of the Bax/Bcl-2 ratio, increased cytochrome c release, and an increase in active caspase-3. Neither niacin nor citicoline at low doses ameliorated these changes. High-dose monotherapies partially preserved PhNR and PERG, limited RGC death, and reduced oxidative, inflammatory, and apoptotic markers. Strikingly, combining niacin (either dose) with low-dose citicoline fully restored PhNR and PERG amplitudes and preserved RGC density to control levels while normalizing Nrf-2, HO-1, phospho-NF-κB, IL-6, GFAP, Bax/Bcl-2, cytochrome c, and caspase-3 to baseline levels. Conversely, pairing either dose of niacin with high-dose citicoline failed to rescue retinal function or structure and only partly reduced molecular stress markers. To date, no clear explanation has been provided for the lack of combined efficacy when citicoline is given at a high dose [104].

These data suggest that niacin and citicoline target complementary mitochondrial and membrane-stabilizing pathways and that their optimal ratio is critical to achieve maximal neuroprotection in hypertensive glaucoma. By attenuating oxidative stress and inflammation upstream of the intrinsic apoptotic cascade, the combination preserves RGCs’ physiology and viability despite persistent IOP elevation. Limitations include the short two-week disease window, supraphysiological supplement doses required in mice, and incomplete knowledge of supplement bioavailability and ocular pharmacokinetics. Nevertheless, these results provide a strong rationale for clinical studies evaluating niacin–citicoline combinations as adjuvant neuroprotective therapy in glaucoma patients [104] (Table 10).

## 7. Citicoline, Homotaurine, Vitamin B3, and Pyrroloquinoline Quinone

The neuroprotective role of citicoline and homotaurine eventually combined with vitamin B3 and pyrroloquinoline quinone has recently been investigated (Table 10).

In a multicenter, prospective, randomized, single-blind crossover trial, 57 patients with POAG, well controlled on topical beta-blockers and prostaglandin analogs (IOP < 18 mmHg) and stable VF (mean deviation <  −12 dB, progression ≤  −1 dB/year), were enrolled. They were randomized to receive either standard topical therapy alone or standard therapy plus a fixed oral combination of 500 mg of citicoline and 50 mg of homotaurine (CIT/HOMO) for four months, with a two-month washout and crossover. The primary outcome, inner retinal function assessed by transient PERG, improved significantly during CIT/HOMO treatment versus topical therapy alone. No significant PERG changes occurred centrally or in steady-state assessments. Secondary end points demonstrated stable visual acuity and mean deviation (MD) on Humphrey 24-2 perimetry alongside a significant reduction in pattern standard deviation (PSD), indicating enhanced field reliability. IOP remained unchanged with adjunctive CIT/HOMO. Vision-related quality of life, measured via the NEI-VFQ-25, showed a significant gain in vision-specific dependency, with non-significant improvements in other subscales. These data indicate that adding once-daily CIT/HOMO to standard IOP-lowering therapy selectively enhances RGCs’ electrophysiological function and may improve VF stability and patient-reported dependency without altering IOP [70].

The adjunction of vitamin E to the combination of citicoline and homotaurine has also been studied. In a multicenter, observational, short-term crossover study, 109 patients with mild POAG and stable, well-controlled IOP (<18 mmHg) were randomized to receive either standard topical IOP-lowering therapy alone or with the addition of a once-daily fixed combination of 500 mg of citicoline, 50 mg of homotaurine, and 12 mg of vitamin E (CIT/HOMO/VITE) for four months. Treatments were then crossed over for an additional four months. Over each four-month treatment period, IOP and SAP-derived VF indices (MD and PSD) remained unchanged. Contrast sensitivity significantly improved during CIT/HOMO/VITE intake. Vision-related quality of life improved with supplementation as the glaucoma-related quality of life (GQL-15) score decreased (indicating better quality of life). Crossover analysis confirmed a significant treatment effect on the Spaeth–Richman SPARCS test and GQL-15 without a significant sequence (carryover) effect. These findings suggest that adding CIT/HOMO/VITE to conventional topical therapy selectively enhances binocular contrast sensitivity and vision-related quality of life in patients with early glaucoma while maintaining stable IOP and VF performance [105].

In a prospective, randomized, single-blind crossover trial, 40 patients with POAG and well-controlled IOP on topical therapy were assigned to two sequences. Group A received four months of standard IOP-lowering drops alone followed by four months of adjunctive once-daily doses of 500 mg of citicoline, 50 mg of homotaurine, and 12 mg of vitamin E (CIT/HOMO/VITE), while Group B received the reverse order. Throughout both treatment phases, the mean IOP, optic nerve head cup-to-disk ratio on OCT, and Humphrey 30-2 MD remained statistically unchanged. In contrast, PERG amplitudes, reflecting inner retinal ganglion cell function, improved significantly during supplementation. At T1, subjects receiving CIT/HOMO/VITE exhibited a rise in the N35–P50 amplitude and in the P50–N95 amplitude. After crossover, those who newly added CIT/HOMO/VITE at T2 showed analogous gains in N35–P50 and P50–N95. Conversely, PERG amplitudes reverted toward baseline when supplementation was withdrawn. Mixed-model crossover analysis confirmed a highly significant treatment effect on both N35–P50 and P50–N95 amplitudes with no evidence of carryover. No serious adverse events occurred, and IOP remained stable. These findings suggest that four months of oral CIT/HOMO/VITE supplementation can selectively enhance electrophysiological markers of retinal ganglion cell activity in early-stage glaucoma. This benefit occurs without altering IOP, optic nerve morphology, or standard perimetric indices and supports a potential neuroprotective role that warrants longer-term controlled studies [106].

In a recent multicenter, randomized, single-blind crossover trial, 40 patients with POAG, well controlled on once-daily prostaglandin analog monotherapy (IOP < 18 mmHg) and exhibiting early to moderate VF defects, were enrolled. They completed two consecutive four-month periods without washout, receiving either 800 mg of citicoline (CIT800) or a fixed combination of 500 mg of citicoline, 50 mg of homotaurine, 54 mg of vitamin B3, and 5 mg of pyrroloquinoline quinone (CIT/HOMO/B3/PQQ). Retinal ganglion cell function was assessed by PERG at baseline, four months, and eight months. Visual acuity, Humphrey 24-2 perimetry, IOP, and vision-related quality of life (NEI-VFQ-25) were evaluated at each visit. All participants completed both phases without serious adverse events. Both CIT800 and CIT/HOMO/B3/PQQ improved PERG P50 and N95 amplitudes and shortened latencies compared to baseline. However, crossover analysis demonstrated superior neuromodulatory efficacy for CIT/HOMO/B3/PQQ. Specifically, this regimen reduced central P50, inferior-field P50, and inferior N95 latencies and increased superior N95 amplitude relative to CIT800. Neither visual acuity nor IOP differed between treatments, and both regimens maintained stable MD and PSD on perimetry. Quality of life improved significantly during CIT/HOMO/B3/PQQ supplementation, with higher NEI-VFQ-25 total scores and positive effects observed in general health, color vision, and peripheral vision. These findings indicate that, in early glaucoma stabilized on IOP-lowering therapy, adjunctive CIT/HOMO/B3/PQQ more effectively enhances electrophysiological markers of RGCs’ activity and patient-reported visual functioning than high-dose citicoline alone without affecting IOP or standard perimetric indices [107].

Although promising, further studies are needed to confirm long-term structural and functional benefits and to validate potential quality-of-life improvements from this multi-compound neuromodulator.

## 8. Nicotinamide and Pyruvate

Nicotinamide and pyruvate are essential compounds involved in cellular metabolism and energy production. Together, these compounds support cellular energy balance, reduce oxidative stress, and enhance metabolic efficiency. Their combined use is being investigated for therapeutic potential in conditions such as neurodegeneration, metabolic disorders, and mitochondrial dysfunction, highlighting their importance in maintaining cellular health (Table 10).

In a phase 2, randomized, double-blind, placebo-controlled trial, 32 of 42 enrolled participants with treated moderate open-angle glaucoma completed the study. Patients were assigned in a 2:1 ratio to receive escalating oral doses of nicotinamide plus pyruvate or a matched placebo over a median follow-up of 2.2 months. The primary endpoint, the number of VF test locations showing sensitivity improvements beyond normal variability, was significantly greater in the treatment group than in the placebo group. Logistic mixed-effects modeling indicated that intervention tripled the odds of pointwise improvement independent of baseline sensitivity. Among secondary VF indices, the PSD slope improved significantly, whereas MD and visual field index (VFI) slopes did not differ significantly. However, more eyes in the treatment arm achieved global MD and VFI gains exceeding the 75th percentile of the cohort distribution. No serious adverse events occurred; mild gastrointestinal discomfort was reported in both groups and did not necessitate discontinuation, except in one treated participant. Structural evaluation by OCT showed no significant change in RNFL thickness during the short study period, and cognitive screening scores remained stable. These findings demonstrate that high-dose nicotinamide combined with pyruvate is safe and yields short-term functional enhancements in glaucomatous visual fields, supporting further investigation of NAD^+^-boosting, bioenergetic therapies for long-term neuroprotection in glaucoma [108].

In this retrospective case series of 16 patients with moderate to advanced primary open-angle glaucoma managed at Stanford’s Byers Eye Institute, Khatib et al. [109] evaluated the real-world tolerability and potential efficacy of oral nicotinamide, with or without pyruvate, as an adjunct to standard IOP-lowering therapy over a mean follow-up of 16.1 ± 2.5 months. Half of the cohort (8/16) took a combination of nicotinamide and pyruvate, while the remainder received nicotinamide alone. Overall adherence was high: 81.2 percent (13/16) continued supplementation through the most recent follow-up, and only three patients (18.8 percent) discontinued, citing gastrointestinal discomfort or skepticism regarding clinical benefit. Objective measures of visual function and structure showed stability rather than deterioration during the supplementation period: best-corrected visual acuity, intraocular pressure, global RNFL thickness, and ganglion cell complex thickness remained unchanged from baseline. Although not reaching statistical significance, there was a favorable trend toward slowed global VF MD progression after supplementation began, suggesting a possible neuroprotective effect. No serious adverse events were reported. These preliminary results suggest that high-dose nicotinamide, with pyruvate in selected cases, is well tolerated in a real-world glaucoma population and may help stabilize RGC-related structure and function over the medium term. Ongoing analyses are exploring localized VF changes, correlations with systemic and ocular mitochondrial biomarkers, optic nerve head perfusion metrics, and electrophysiological markers of ganglion cell health. The aim is to clarify the mechanisms behind these observations and identify patient subgroups most likely to experience benefits.

Despite these encouraging findings, optimal dosing, long-term safety, and meaningful functional outcomes must be confirmed in larger trials.

## 9. Conclusions

The management of glaucoma has traditionally focused on lowering IOP, yet many patients still experience progressive vision loss despite reaching target IOP levels. Neuroprotective treatments represent a promising approach by targeting multiple molecular and cellular mechanisms involved in glaucomatous damage, including oxidative stress, inflammation, mitochondrial dysfunction, and excitotoxicity. Evidence suggests that, rather than relying on a single therapeutic target, a combination of neuroprotective agents may yield synergistic effects, providing broader and more effective protection of the optic nerve.

The potential benefit of multiple neuroprotective treatments lies in their ability to act on different pathways of glaucoma pathophysiology. For instance, agents that enhance GABAergic signaling can reduce excitotoxic neuronal damage, while neurosteroids may protect RGCs through anti-apoptotic and anti-inflammatory mechanisms. Antioxidants help counteract oxidative stress, and mitochondrial stabilizers support cellular energy metabolism. When used together, these agents may complement each other, leading to greater neuroprotection than monotherapy.

Moreover, a multifaceted neuroprotective strategy is consistent with the complex nature of glaucoma, which involves not only mechanical and vascular factors but also a cascade of biochemical events leading to neuronal death. Personalized combinations of neuroprotective agents, tailored to an individual’s disease profile, may enhance treatment efficacy and slow disease progression more effectively than current standard approaches.

In conclusion, integrating multiple neuroprotective treatments into glaucoma management has the potential to transform the therapeutic landscape. By addressing the disease at a neurobiological level, such strategies have the potential to preserve visual function, improve quality of life, and reduce the burden of irreversible blindness. Ongoing research and clinical trials will be critical for determining optimal combinations, dosages, and treatment timing to fully harness the benefits of this approach.

## Figures and Tables

**Table 1:** Summary of reviewed preclinical and clinical studies on citicoline.

Molecule	First Author	Main Findings
Citicoline	Virno et al., 2000 [17]	A dose of 1 g/day of intramuscular citicoline for 15 days repeated every 6 months prevented the progression of perimetric deficits in glaucomatous patients.
	Giraldi et al., 1989 [18]	A dose of 1 g/day of intramuscular citicoline for 10 days led to significant perimetric improvement in 75% of eyes that remained stable for at least 3 months.
	Matteucci et al., 2014 [19]	Citicoline (100 μM) was able to counteract neuronal cell damage in retinal cultures by decreasing proapoptotic effects and contrasting synapse loss.
	Parisi et al., 1999 [20]	A dose of 1 g/day of intramuscular citicoline for 60 days improved cortical and retinal responses (PERG and VEP) and sustained functional benefits.
	Parisi et al., 2005 [21]	A dose of 1 g/day of intramuscular citicoline via repeated treatment significantly improved retinal and cortical responses in glaucoma patients, as measured by VEPs and PERGs. Effects were sustained during washouts and enhanced with long-term treatment, suggesting citicoline’s potential as a neuroprotective complement to standard glaucoma therapy.
	Parisi et al., 2008 [22]	A dose of 1600 mg/die oral or 1000 mg/die intramuscular citicoline treatment improved retinal function and visual pathway conduction in glaucoma patients. Effects regressed after washout, but long-term treatment stabilized or improved visual function, suggesting citicoline’s neuroprotective potential in glaucoma management.
	Parisi et al., 2015 [23]	Topical citicoline (0.2 g, 3 drops/day) over a 4-month period induced an enhancement of retinal bioelectrical responses (PERG).
	Roberti et al., 2014 [24]	The experimental phase showed the ability of citicoline 1% and 2% eye drops to reach the vitreous and the retina. The clinical phase showed a positive trend of perimetric parameters in individual eyes treated with citicoline 1% eye drops.
	Sahin et al., 2022 [26]	Increased RNFL thickness was observed after 3 months of 250 mg/day oral citicoline treatment in POAG patients.
	Rossetti et al., 2023 [27]	Enhanced quality of life was observed in POAG patients using 500 mg/day oral citicoline.
	Prinz et al., 2023 [28]	This systematic review of 10 studies (424 patients) found no significant effects of citicoline on IOP, VF, RNFL thickness, or PERG amplitude in glaucoma, indicating insufficient evidence for its role in slowing disease progression.

PERG: pattern electroretinogram; VEP: visual evoked potential; RNFL: retinal nerve fiber layer; POAG: primary open-angle glaucoma; IOP: intraocular pressure; VF: visual field.

**Table 2:** Summary of reviewed preclinical and clinical studies on coenzyme Q10.

Molecule	First Author	Main Findings
Coenzyme Q10	Russo et al., 2008 [31]	In an animal model of retinal ischemia, CoQ10 conferred retinal protection by mitigating elevated extracellular glutamate-induced excitotoxicity.
	Davis et al., 2017 [32]	Topical CoQ10/TPGS demonstrated significant neuroprotection in glaucoma models, reducing RGCs apoptosis in vitro and in vivo, as shown by DARC imaging and Brn3a histological analysis.
	Lee et al., 2014 [33]	Oral CoQ10 protects RGCs against oxidative stress by modulating the Bax/Bad-mediated mitochondrial apoptotic pathway and prevents mitochondrial alteration by preserving Tfam protein expression in ischemic retina.
	Parisi et al., 2014 [36]	Topical CoQ10/TPGS at a dose of 2 drops/day shows a beneficial effect on the inner retinal function (PERG) in OAG with a consequent enhancement of visual cortical responses (VEP).
	Ekicier Acar et al., 2020 [34]	In a rat mechanical optic nerve injury model, topical CoQ10 + vitamin E preserved retinal ganglion cells, reduced astroglial activation and microglial response, increased anti-apoptotic protein Bcl-xL, and maintained mitochondrial Tfam expression, indicating potential neuroprotection for glaucoma therapy.
	Ozates et al., 2019 [35]	In pseudo-exfoliative glaucoma, one-month topical CoQ10 + Vit.E significantly reduced aqueous humor superoxide dismutase levels compared to untreated cases, while pseudo-exfoliation syndrome showed even lower levels. Malondialdehyde remained unchanged across groups, suggesting selective modulation of oxidative stress without affecting lipid peroxidation.
	Dogan et al., 2025 [37]	In POAG, adjunctive CoQ10 + Vit.E with standard therapy improved VEP parameters, preserved visual fields in most patients, and slowed ganglion cell loss compared to controls, while both groups showed RNFL thinning, indicating potential neuroprotective benefits on retinal structure and cortical visual responses.
	Ju et al., 2018 [39]	Ubiquinol supplementation protects RGCs in ischemic mouse retina by reducing astroglial and microglial activation, modulating the Bax/Bad/Bcl-xL apoptotic pathway, and preventing caspase-3-mediated apoptosis, indicating therapeutic potential against elevated intraocular pressure-induced retinal degeneration.
	Edwards et al., 2020 [40]	Ubiquinol supplementation enhances RGCs’ survival, inhibits BAX activation, and increases TFAM and OXPHOS complex II expression in mouse models of glaucoma and oxidative stress, preserving visual function and demonstrating therapeutic potential against glaucomatous neurodegeneration.
	Martucci et al., 2019 [41]	CoQ10 100 mg Miniactives^®^ bioavailability was assessed in 24 healthy adults showing effective plasma concentration with once or twice daily dosing. Miniactives^®^ technology enabled sustained CoQ10 levels without adverse effects, suggesting potential for consistent antioxidant support in chronic conditions requiring elevated CoQ10 levels.

PERG: pattern electroretinogram; VEP: visual evoked potential; RNFL: retinal nerve fiber layer; POAG: primary open-angle glaucoma; CoQ10: coenzyme Q10; RGCs: retinal ganglion cell; TPGS: α–tocopherol polyethylene glycol succinate; DARC: detection of apoptotic retinal cell; Vit.E: vitamin E.

**Table 3:** Summary of reviewed preclinical and clinical studies on pyruvate.

Molecule	First Author	Main Findings
Pyruvate	Choi et al., 2010 [46]	Ethyl pyruvate shows neuroprotective effects in Parkinson’s disease models by preventing dopaminergic neuron death, reducing inflammation, and restoring extracellular signal-regulated kinase phosphorylation. Ethyl pyruvate suppressed toxin-induced cell death in mice and SH-SY5Y cells.
	Famili et al., 2013 [48]	Ethyl pyruvate is a well-tolerated antioxidant in human trabecular meshwork cells, enhancing survival under oxidative stress when given before and during exposure. However, alone, it does not boost endogenous defense, suggesting continuous delivery may be necessary for effective glaucoma therapy.
	Hegde et al., 2008 [49]	This study examined oxidative stress-induced damage in mouse retinal tissue and tested pyruvate’s protective effects. ROS exposure reduced glutathione and increased malonaldehyde, indicating damage. Pyruvate significantly attenuated these effects, suggesting that its dual role as a reactive oxygen species scavenger and metabolic supporter may help prevent age-related retinal degeneration.
	Harder et al., 2020 [50]	Early glaucoma involves intraocular pressure-dependent metabolic disruption, including reduced pyruvate and elevated retinal glucose. Pyruvate supplementation and mTOR inhibition (via rapamycin) protected against neurodegeneration in glaucoma models, highlighting metabolism-targeted therapies as promising, translatable strategies for preventing glaucoma-related retinal ganglion cell loss.

**Table 4:** Summary of reviewed preclinical and clinical studies on nicotinamide.

Molecule	First Author	Main Findings
Nicotinamide	Airhart et al., 2017 [57]	Oral NR was well tolerated and significantly increased blood levels of NR and NAD^+^ in healthy volunteers, with NAD^+^ doubling by Day 9. These findings suggest NR’s potential as a treatment for mitochondrial dysfunction-related conditions.
	Williams et al., 2017 [58]	Mitochondrial dysfunction and declining NAD^+^ levels precede neurodegeneration in glaucoma-prone mice. Oral nicotinamide (vitamin B3) and Nmnat1 gene therapy preserved neuronal health, preventing glaucoma in 93% of treated eyes. These findings support NAD^+^-boosting therapies for glaucoma and age-related neurodegenerative diseases.
	Williams et al., 2018 [59]	Age-related NAD decline contributes to early mitochondrial dysfunction in retinal ganglion cells, increasing vulnerability to glaucoma. Nicotinamide treatment significantly reduced glaucoma risk, prevented optic nerve damage, and preserved axons. It also stabilized expression of NAD-producing enzymes, supporting nicotinamide’s strong neuroprotective potential in glaucoma therapy.
	Hui et al., 2020 [61]	In this randomized, double-masked clinical trial, nicotinamide significantly improved inner retinal function in glaucoma patients, shown by enhanced PhNR parameters and visual field trends compared to placebo.

NR: nicotinamide riboside; NAD^+^: nicotinamide adenine dinucleotide; PhNR: photopic negative response.

**Table 5:** Summary of reviewed preclinical and clinical studies on pyrroloquinoline quinone.

Molecule	First Author	Main Findings
Pyrroloquinoline quinone	Harris et al., 2013 [64]	Oral pyrroloquinoline quinone affects mitochondrial and neurological function. In a crossover study, PQQ intake altered antioxidant potential and reduced inflammation markers in humans, with no change in clinical indices. Urinary metabolites suggested enhanced mitochondrial activity, linking animal and human responses.

PQQ: pyrroloquinoline quinone.

**Table 6:** Summary of reviewed preclinical and clinical studies on homotaurine.

Molecule	First Author	Main Findings
Homotaurine	Spalletta et al., 2016 [67]	Homotaurine supplementation in individuals with amnestic mild cognitive impairment reduced hippocampal and temporal lobe atrophy over one year and improved short-term episodic memory. Neuroimaging showed decreased volume loss in key brain areas, suggesting a neuroprotective effect. Further research is needed to understand its underlying mechanisms.

**Table 7:** Summary of reviewed preclinical and clinical studies on berberine.

Molecule	First Author	Main Findings
Berberine	Campisi et al., 2011 [72]	*Berberis aetnensis* root extract, rich in berberine, counteracts glutamate-induced excitotoxicity in rat astrocyte cultures by restoring oxidative balance and tissue transglutaminase (TG2) levels. It also reduces protein misfolding, mitochondrial damage, and neurodegeneration, suggesting potential as a natural therapeutic for excitotoxicity-related neurological disorders.
	Ye et al., 2024 [73]	Berberine protects against light-induced retinal degeneration by preserving retinal structure, reducing photoreceptor apoptosis, and suppressing inflammation. It downregulates the P2X7 receptor, which mediates oxidative stress and cell death. In knockout mice, berberine showed enhanced protection, suggesting P2X7R involvement in its neuroprotective effects against retinal damage.
	Fang et al. 2022 [74]	Berberine protects retinal RGCs in diabetic retinopathy by enhancing cell survival, reducing apoptosis, and improving visual function. Its effects involve the upregulation of GABA-alpha receptors and PKC-α. Blocking this pathway with SR95531 reverses BBR’s benefits, highlighting its therapeutic potential via the GABAAR/PKC-α mechanism.

RGCs: retinal ganglion cells; GABA: gamma-aminobutyric acid; PKC-α: protein kinase C alpha.

**Table 8:** Summary of reviewed preclinical and clinical studies on gamma-aminobutyric acid.

Molecule	First Author	Main Findings
Gamma-aminobutyric acid	Bailey et al., 2014 [76]	A genome-wide study identified several KEGG pathways associated with POAG, notably butanoate metabolism, which relates to GABA and acetyl-CoA metabolism. This pathway was also linked to normal-pressure glaucoma, highlighting its central role in glaucoma pathogenesis and potential for novel therapeutic targeting.
	Bang et al., 2023 [77]	Using magnetic resonance techniques, this study found that both GABA and glutamate levels in the visual cortex decline with glaucoma severity. Notably, reduced GABA—but not glutamate—predicts decreased neural specificity. This suggests that GABA loss disrupts visual processing and may be a promising therapeutic target for glaucoma-related brain changes.
	Zhou et al., 2019 [78]	Activating 5-HT1A receptors protects RGCs in glaucoma by enhancing GABAergic signaling via the cAMP-PKA pathway. 8-OH-DPAT increased GABA release and RGC viability, effects blocked by GABAA or 5-HT1A antagonists, revealing a potential therapeutic mechanism targeting serotonergic modulation in glaucoma.

RGCs: retinal ganglion cells; GABA: gamma-aminobutyric acid; KEGG: Kyoto Encyclopedia of Genes and Genomes; POAG: primary open-angle glaucoma; 8-OH-DPAT: 8-hydroxy-2-(di-n-propylamino) tetralin; cAMP-PKA: cyclic adenosine-protein kinase A.

**Table 9:** Summary of reviewed preclinical and clinical studies on promising neuroprotective molecules.

Molecule	First Author	Main Findings
Allopregnanolone	Ishikawa et al., 2014 [79]	Ex vivo rat retinas exposed to increasing hydrostatic pressure showed elevated allopregnanolone production. This elevation was suppressed by NMDA receptor and 5α-reductase inhibitors. Exogenous allopregnanolone reduced axonal swelling via GABAA receptors, suggesting its therapeutic potential for protecting against pressure-induced retinal damage in glaucoma.
Riluzole	Yildiz et al., 2024 [80]	Twenty-eight Wistar rats were divided into 4 groups to study glaucoma and riluzole’s effects. Riluzole reduced IOP and slightly decreased MMP-2 and MMP-9 expression. Histopathological changes showed no significant differences. Both corn oil + DMSO and riluzole reduced gene expression compared to the glaucoma-only group.
Epigallocatechin gallate	Gasiunas et al., 2022 [81]	A study with 43 volunteers showed that green tea and EGCG extracts significantly reduced IOP compared to the placebo, potentially benefiting individuals with elevated IOP or those at risk of glaucoma.

GABAA: gamma-aminobutyric acid; NMDA: N-methyl-D-aspartate; IOP: intraocular pressure; DMSO: dimethyl sulfoxide; EGCG: epigallocatechin gallate.

**Table 10:** Summary of reviewed preclinical and clinical studies on combined use of neuroprotective molecules.

Molecule	First Author	Main Findings
Citicoline, Coenzyme Q10, and Vitamin B3	Mastropasqua et al., 2022 [101]	The efficacy of citicoline, CoQ10, and vitamin B3, with their fixed combination, was assayed after the exposure of hypothalamic cells to hydrogen peroxide. The combination showed biocompatibility, reduced inflammatory markers IL-6 and TNFα, decreased dopamine degradation, and increased neurotrophin BDNF expression, highlighting its neuroprotective potential.
	Di Simone et al., 2024 [102]	This study evaluated citicoline, CoQ10, and their combination for protecting rat astrocytes against oxidative stress. All treatments were biocompatible and reduced hydrogen peroxide-induced apoptosis and inflammation by modulating key gene expressions. The combined treatment showed stronger neuroprotective and antioxidant effects than individual compounds.
	Matamoros et al., 2025 [103]	This study examined the combined effect of citicoline and CoQ10 on glial activation in a mouse model of OHT. Oral treatment reduced IOP and decreased macroglial and microglial activation in the retina and visual pathway, suggesting neuroprotective effects against OHT-induced inflammation.
Citicoline and Vitamin B3	Melecchi et al., 2023 [104]	In a mouse model of ocular hypertension, the combination of niacin and citicoline preserved about 50% of RGCs, improved electroretinographic responses, and reduced oxidative and inflammatory markers. While the results show strong neuroprotective effects, further studies are needed to assess clinical relevance and human applicability.
Citicoline, Homotaurine, Vitamin B3, and Pyrroloquinoline Quinone	Rossi et al., 2022 [70]	This study evaluated the neuroprotective effects of oral citicoline and homotaurine in glaucoma patients with controlled IOP. After 4 months, patients showed significant improvements in RGC function, as evidenced by enhanced PERG wave amplitudes and shorter peak times.
	Marino et al., 2020 [105]	This study assessed the effects of a fixed combination of citicoline, homotaurine, and vitamin E on patients with mild POAG. While IOP and VF remained stable, contrast sensitivity and quality of life significantly improved with supplementation. The results support this combination as a beneficial adjunct to standard glaucoma therapy.
	Cornelio et al., 2021 [106]	This randomized crossover study evaluated the effect of a citicoline, homotaurine, and vitamin E combination on RGCs’ function in POAG patients with controlled IOP. PERG amplitude improved significantly during supplementation, indicating enhanced inner retinal function, without affecting IOP, VF, or optic disk parameters.
	Rossi et al., 2025 [107]	In early glaucoma patients, oral supplementation with a fixed combination of citicoline, homotaurine, vitamin B3, and PQQ significantly improved PERG parameters and quality of life compared to citicoline alone. The combination showed greater neuromodulatory benefits, likely via enhanced mitochondrial function, with no changes in IOP or visual acuity observed.
Nicotinamide and pyruvate	De Moraes et al., 2022 [108]	In this randomized, placebo-controlled trial, oral nicotinamide and pyruvate significantly improved VF test locations in glaucoma patients over a short period. While VF global indices showed limited changes, the treatment group outperformed the placebo in pattern standard deviation improvements.
	Khatib et al., 2024 [109]	In this real-world case series of 16 POAG patients, oral nicotinamide and pyruvate supplementation was well tolerated over 16 months, with 81.2% of patients continuing treatment. No significant structural or functional changes were observed, though a trend toward slower VF deterioration emerged. Patients expressed strong interest in adjunctive neuroprotection alongside standard IOP-lowering therapy.

TNFα: tumor necrosis factor α; BDNF: brain-derived neurotrophic factor; OHT: ocular hypertension; IOP: intraocular pressure; CoQ10: coenzyme Q10; RGCs: retinal ganglion cells; PERG: pattern electroretinogram; POAG: primary open-angle glaucoma; VF: visual field; PQQ: pyrroloquinoline quinone.

## Data Availability

All data is available on PubMed.
